# Perspective: Is It Time to Revise the Current Nutrient Requirements for Infant Formulas Principally Established in 1980?

**DOI:** 10.1016/j.advnut.2023.02.006

**Published:** 2023-03-05

**Authors:** Steven A. Abrams, Erynn M. Bergner

**Affiliations:** 1 Department of Pediatrics, Dell Medical School at the University of Texas, Austin, TX, United States; 2 Department of Pediatrics, University of Oklahoma College of Medicine, Oklahoma City, OK, United States

**Keywords:** infant formula, iron requirements, fatty acids, protein requirements, premature infants

## Abstract

Because of the production of nutrient-deficient infant formulas (IFs), the United States Congress passed regulations on the composition and production of IF, referred to as the Infant Formula Act (IFA), in 1980, which was amended in 1986. More detailed FDA rules have been created since then, specifying the ranges or minimum intakes of nutrients and providing details for the safe production and evaluation of infant formulas. Although generally effective in ensuring safe IF, recent events have made it clear that a re-evaluation of aspects of all the nutrient composition regulations for IF is needed, including consideration of adding requirements related to bioactive nutrients not mentioned in the IFA. We propose that, as principal examples, the requirement for iron content needs to be re-evaluated and that DHA and AA should be considered for addition to the nutrient requirements after scientific review by a panel such as those established by the National Academies of Sciences, Engineering, and Medicine. Additionally, there is no specific requirement in current FDA regulations for the energy density of IF, and this should be added alongside potential revisions of the protein requirement. It would also be ideal to have specific FDA rules on nutrient intakes for premature infants as these are exempted from the specific nutrient regulations of the amended IFA.

**Statement of significance**

We propose that the IFA of 1980, upon which current FDA regulations for the nutrient content of IF in the United States are largely based, does not represent the most updated science and would benefit from a comprehensive, independent review. We provide specific examples of nutrient-related issues to be re-evaluated and potentially revised.

## Introduction

Although introduced in the 19th century, infant formulas (IFs) only became widely used in the middle of the 20th century. Nutrient recommendations were provided by organizations including the American Academy of Pediatrics (AAP) and others [1,2], but these were generally incomplete, and at times manufacturers made decisions without a full understanding of infant physiology. Specifically, the production and use of a formula with low sodium chloride concentration in the late 1970s led to adverse infant health outcomes prompting a Congressional legislative act, the Infant Formula Act (IFA) of 1980, that mandated details of FDA regulation of IF and, uniquely, the nutrient composition of IF [3,4].

Changes were made to the IFA in 1986, including comprehensive nutrient testing and requiring that regulations be established for quality control as needed. Additional specific rules related to formula production were developed over time and most recently finalized in 2014 [5]. In particular, FDA established rules for quality factors of normal physical growth and the biological quality of protein in the formula as evidenced by a growth monitoring study and a protein efficiency rat bioassay, respectively. They further established comprehensive good manufacturing practices before a new formula may be registered by the FDA and marketed in the United States.

It is important to note that the FDA has the legal authority to alter the regulations regarding the levels of nutrients in formulas without new congressional legislation, although this has not broadly been done, with the notable exception of the addition of selenium to the required nutrient list in 2016 [6]. Most other optional ingredients, especially bioactive ingredients, are electively added to IFs using the GRAS notification process but are not found in all IFs.

The current nutrient standards of the FDA for IF are shown in Table 1 [7]. Notably, there are only a few nutrients for which both upper and lower bounds are provided, and these are often very wide ranges. No requirements for bioactives, including FAs such as DHA and AA, oligosaccharides, and other ingredients currently found in many marketed IFs, are contained within these regulations. The recent IF shortages have led to the need to import formulas rapidly worldwide [8]. The nutrient contents for these are principally based on European or Australian regulations and, in some cases, differ from those of the FDA. The FDA has reviewed these and considers them acceptable for the United States use during the formula shortage, but this has led to the potential for public confusion in the measurement of formula and water during preparation (e.g., use of mL instead of ounces) as well as new regulations based on these variations by the Special Supplemental Nutrition Program for Women, Infants, and Children (WIC) [9].

**TABLE 1:** Nutrient requirements of infant formulas as outlined by the Infant Formula Act and revised by the FDA^7^

	Minimum^1^	Maximum^1^
Nutrients:		
Protein, g	1.8^2^	4.5^2^
Fat, g	3.3	6.0
LA, mg	300	-
Vitamins:		
A, IU	250	750
D, IU	40	100
E, IU	0.7	-
K, mcg	4	-
B_1_ (thiamine), mcg	40	-
B_2_ (riboflavin), mcg	60	-
B_6_ (pyridoxine), mcg	35	-
B_12_, mcg	0.15	-
C (ascorbic acid), mg	8	-
Niacin, mcg	250	-
Folic acid, mcg	4	-
Pantothenic acid, mcg	300	-
Biotin, mcg	1.5^3^	-
Choline, mg	7^3^	-
Inositol, mg	4^3^	-
Minerals:		
Calcium, mg	60^4^	-
Phosphorus, mg	30^4^	-
Magnesium, mg	6	-
Iron, mg	0.15	3.0
Iodine, mcg	5	75
Zinc, mg	0.5	-
Copper, mcg	60	-
Manganese, mcg	5	-
Selenium, mcg	2	7
Sodium, mg	20	60
Potassium, mg	80	200
Chloride, mg	55	150

LA, linoleic acid.

1 Stated per 100 kcal.

2 The source of proteins should be at least nutritionally equivalent to casein. If the quality is less than casein, the minimum amount of protein should be increased proportionately to compensate. No protein with a biological quality <70% of casein should be used.

3 Required to be in this amount only in formulas that are not milk-based.

4 The calcium to phosphorus ratio must be no <1.1 nor >2.0.

The globalization of the production and distribution of IF leads to a need to move toward modernizing the regulations for nutrient composition of IF and considering having the United States adopt global nutrient standards when possible. It also provides an opportunity to look closely at formulas defined as exempt from the requirements for routine term IFs, especially those intended for premature infants, and consider including some of these formulas within a revision of FDA regulations on the nutrient content of IF [10].

We discuss herein a few specifics of the current nutrient regulations and how they might be revised in keeping with more recent science and global standards. This approach would ideally be undertaken as a comprehensive assessment by an independent panel, such as one established by the National Academies of Sciences, Engineering, and Medicine (NASEM), for all the nutrients (and other substances, such as emulsifiers) in IF, both for preterm and full-term infants. These examples will provide information for understanding some of the issues involved.

### Definition of Infant Formula

Current FDA regulations included in the IFA define IF as “food which purports to be or is represented for special dietary use solely as a food for infants by reason of its simulation of human milk or its suitability as a complete or partial substitute for human milk” [4]. Although human milk serves as the gold standard of infant nutrition, multiple considerations prevent IF from fully mimicking human milk. Differences in bioavailability and alterations to the nutritional value that occur in processing often require the nutrient content of formula to differ from that of human milk to achieve the same biologic effect. These variations, however, tend to be broad and not uniformly applicable. For example, calcium absorption may be affected by fat sources, potentially decreased with the use of palm olein, and increased with Sn-2 sources of oil [11,12].

We propose that amendments to the IFA definition include clarifications that the clinical outcomes achieved by human milk are the target of IF rather than the individual components themselves. This includes assessments of bioactives that are added to IFs and clinical outcomes of long-term disease prevention, such as asthma. Recognition of the differences between IF and human milk will both ensure a focus on infant health and better align with the WHO’s code of marketing of breastmilk substitutes by limiting false claims of equivalence of formula and human milk [13]. In doing so, it is also time to implement long-term evaluation postmarketing as suggested by the Institute of Medicine report on IF, although it is recognized that long-term evaluation has limited sensitivity for many outcomes [14]. As bioactive components increasingly enter the conversation, a framework that ensures both safety and efficacy of included components is paramount to optimizing infant outcomes.

## Nutrients

### Iron

#### Background and history of iron guidelines in infant formula

The original IFA mandated a minimum of 0.15 mg per 100 kcal (approximately 1 mg/L) for infant formula. An upper amount of 3.0 mg/100 kcal (approximately 20 mg/L) was also established in later years. As sold in the United States, IFs were not initially iron-fortified, but in 1959 the first iron-fortified formula was marketed (Similac with Iron) containing 12 mg/L as prepared [3]. There were no research data specific to the upper or lower levels, and this quantity was likely selected based on the estimation of the iron bioavailability as added to the formula. In the 1960s–1980s, there was both pediatric and public belief that iron-fortified formulas led to gastrointestinal upset and constipation, and many formulas were produced that were low iron. In 1969 the AAP indicated that iron intake should be 1 mg/kg/d by 3 mo of age to a maximum of 15 mg/d in the first year of life and strongly discouraged using low-iron formulas [2].

The vast majority of infant formula produced in the United States for full-term infants contains the same 12 mg/L iron content introduced in 1959. There do not appear to be any IF marketed above that level. Beginning in late 2021 and continuing with the importation of formulas related to the shortages, a variety of iron formulas below 12 mg/L have begun to become more widely used in the United States. The FDA has provided additional regulations [7,15] that any formula with an iron content of 1 mg/100 kcal (6.7 mg/L based on usual preparation) or more of iron must be labeled as “with iron” and formulas with less than that have a label indicating the potential need for additional iron. We are unaware of any nonexempt formula produced in the United States for healthy children that are currently labeled as needing additional iron, although some specialized (exempt) products are low iron.

Additional input has come from the USDA via the WIC program indicating that formulas providing <10 mg/L of iron cannot be used for the WIC-contracted IF. These rules were adjusted during the recent formula shortage to 6 mg/L to allow a variety of lower iron formulas imported into the United States to be eligible for coverage as part of WIC.

#### More recent science and regulation

The potential risks of excess iron in infants have recently become clear. These include increased risks of infection and slower growth [16]. A key recent study from Chile also found worse long-term developmental outcomes in infants over 6 mo of age who received formula with 12 mg/L of iron compared to 2 mg/L [17]. A review of the literature by European authorities and scientific groups [18,19] indicates much lower targets, usually 4–8 mg/L of iron in IF. The European Food Safety Authority allows formula for infants up to 6 mo of age as low as 2 mg/L.

These differences led to the current situation where some formulas for full-term infants produced in Europe cannot meet the ordinary WIC standard for iron and are well below the usual United States levels. Although the WIC rules have temporarily been changed, as noted above, a final long-term resolution of these differences has not yet occurred. Pediatricians and others trained to promote high intake levels of iron from formula may be confused about how to deal with possible at-risk groups such as late preterm infants.

We propose that the FDA and WIC regulations for IF need reconsideration and likely revision. There is no need for routine formula iron levels as low as 0.15 mg/100 kcal as is currently allowed. Rather, regulations consistent with the European regulations of 4–8 mg/L should be considered for all formulas. WIC may choose to limit formulas in its programs to 6 mg/L as it is doing under the current special rules or could accept 4 mg/L. Given the relatively higher nutritional risk of the WIC population, the 6 mg/L requirements may be optimal for WIC-approved formulas. Further data are needed, especially as related to the effects of delayed cord clamping on iron requirements. An intake at the maximum of 20 mg/L is also above any possible biological need and likely should be reduced to not more than about 15 mg/L, which allows for a label claim of 12 mg/L with some overage in analyze content as is typical in the production of formulas.

### Fatty acids: DHA and AA

The IFA does not contain any requirements or other regulations for many nutrients that are found in current formulas, which have been evaluated by the FDA and added via the GRAS evaluation process. Largely these bioactives were not available in the 1980s or not available at levels usable for formula production. This includes carnitine, taurine, oligosaccharides, probiotics, lactoferrin, maternal fat globule membranes, nucleotides, and others [20].

One bioactive ingredient that is now found in most IF sold in the United States is DHA. This was allowed by the FDA alongside AA using the GRAS process in 2001 [21]. However, the scientific rationale for using DHA in IFs has been the subject of many studies and reviews and remains controversial, both in terms of the benefits and the ideal amount to include. Lacking specific recommendations on these issues from the AAP, FDA, and most other United States groups, different formula manufacturers have made various decisions regarding this, and there is a fairly large range of DHA and AA content found in formulas sold in the United States.

Recently, standards for DHA in infant formula marketed in Europe after early 2020 were set with minimum values that are above the level typically found in formulas in the United States but consistent with the GRAS notification values allowed by the FDA [22]. This was largely based on reviews of effects on visual and developmental outcomes. These issues are reviewed by Koletzko et al. [23], who also supported recommendations related to the inclusion of AA whenever DHA is provided.

In light of the now increasing use of European formulas in the United States and the lack of any requirement for the addition of DHA and AA in IFs in the United States, it would be important for the United States via NASEM or a similar process to review the literature and the European and Codex Alimentarius recommendations and provide input into DHA and AA provision in infant formula. A NASEM review of macronutrients has been planned, but the details of its review related to DHA are not yet known. We do not make specific recommendations on the outcome of this review, although given its use in the United States marketplace and the current scientific information, we expect that the addition of DHA would be supported by this review along with AA. The specifically chosen range of levels is less clear.

### Protein and energy density

The IFA and current FDA regulations specify a wide range of proteins (1.8–4.5 g/100 kcal or 1.2–3.0 g/dL) and provide no regulations on total energy density [7]. In 1998, the Life Sciences Research Office of the ASN report on nutrient requirements for infant formula recommended a minimum energy density of 63 kcal/dL and protein of 1.7 g/100 kcal for standard term formulas [24]. These exist as the most widely utilized source for IF macronutrient recommendations in the United States. International standards, as outlined by the Codex Alimentarius [25], specify that formulas intended for term infants should contain no <60 kcal and no more than 70 kcal/100 mL. Likewise, Codex Alimentarius standards for protein are set at 1.8g–3.0 g/100 kcal.

Rapid weight gain in infancy has repeatedly been linked to an increased risk of future obesity [[26], [27], [28]]. Meanwhile, formula-fed infants are more likely to demonstrate rapid weight gain than their breastfed counterparts [29,30]. Regulations for energy density range for infant formula that result in infant growth velocities most closely resembling those of breastfed infants when the formula is consumed in a typical ad libitum fashion are needed [31]. Recommendations should be made with caution as these may affect low birthweight, nutritionally at-risk populations.

The European Food Safety Authority recently recommended decreasing the maximum protein for bovine or goat milk-based IFs to 2.5 g/100 kcal and 2.8 g/100 kcal for formulas containing isolated soy protein, citing a lack of physiologic evidence for protein needs in excess of 3.0 g/100 kcal in healthy infants [19]. Although further long-term research is needed, IFs with protein content near the minimum requirements appear to adequately support infant and early childhood growth in healthy term infants [32]. Furthermore, excessive protein intakes in infancy have been associated with more rapid weight gains in infancy and a higher risk of obesity at school age [[33], [34], [35]]. This later metabolic risk is postulated to occur through early alterations in the insulin-like growth factor pathways [27].

Increasing evidence demonstrates that not only protein quantity but also protein quality is an important consideration for infant formula composition. International expert opinion [36] and standards [19,25] agree that the amino acid profile and levels found in human milk should be regarded as the gold standard for infant formula composition. As novel proteins and peptides become at the forefront of consideration as potential bioactive targets (e.g., by providing for functions of amino acids other than as precursors for protein synthesis), it will be important to ensure regulations are in place to maintain the appropriate balance of essential nutrients [20].

We recommend revisions to FDA IF regulations for protein and energy density that both better align with international standards and create safeguards within which the field of formula science can advance. We caution that nutritionally at-risk populations (including late preterm and small for gestational age full-term infants) are also likely to receive formulas intended for healthy term infants. Specific energy and protein requirements of this population and studies that target these infants’ outcomes on lower protein and less energy-dense formula preparations should be considered prior to recommend these populations receive them.

### Preterm and postdischarge formulas

More than 10% of infants worldwide are born preterm [37]. These infants are at increased risk of nutritional deficits because of their shortened gestation. Although national and international mandates rightly allow formulas intended for infants with special healthcare needs to alter their composition to meet the targeted population’s needs (FDA refers to these as exempt formulas), they provide no further specific nutrient regulations on the composition of these formulas, which are then reviewed based on available scientific data by the FDA before registration and subsequent marketing.

Given the large number of affected infants, increased nutrient needs, and difficulties with initiating and maintaining breastfeeding in this population, preterm and postdischarge formulas are prime targets for expanding the nutrient regulatory values in the IFA and international standards alike. Preterm infants require higher energy, protein, iron, DHA, AA, calcium, phosphorus, vitamins, and trace elements than term-born infants [[38], [39], [40]]. Global and national requirements would assist in standardizing nutritional practices for this vulnerable population and pave the way to include additional components as nutrition science advances equitably. Although several groups have made proposals in this regard in recent years, a full evaluation by a neutral scientific organization is needed.

### Other issues

This review has specifically focused on the nutritional composition of IF. It is also clear that the processes by which novel IFs, including those made with vegetable, transgenic human milk proteins, or non-cow’s milk (e.g., goat’s milk) protein sources, are regulated require attention. The current FDA pathways for evaluating and registering novel IF using growth studies have substantial limitations that are not considered here. An independent panel that evaluates nutrient requirements for IF should also evaluate the specific processes and rules related to the registration of formulas in the United States.

One additional difference brought to light by the infant formula shortage is that the labeling requirements of United States formulas differ from that of many internationally manufactured formulas. A substantive reason is that based on Codex regulations, most other countries label formulas with an average nutrient value. The FDA’s primary concern for United States formulas is that they always satisfy minimum requirements during the labeled shelf life period and tolerate label declarations that may be substantially below actual analyzed values at any given time so long as the required levels are present. It would be more informative to healthcare professionals and caregivers if the nutrition content as declared on the label represented very closely the actual typical content of the product rather than a minimum content. Together with modification to the nutrient requirements, the revision of labeling of infant formula would remove 2 impediments to the use of international formulas to meet marketplace shortages.

In summary, our recommendations are shown in Table 2. A reconsideration of the regulations regarding the nutrient content of IF is needed. These revisions should both update regulations to better align with the current body of evidence and create bounds within which innovation in nutrition science can take place. Modernization of the guidelines in the United States is an opportunity to integrate international standards and continue to improve the health and safety of infants going forward.

**TABLE 2:** Summary of key revisions for the Infant Formula Act and subsequent FDA regulations on infant formula composition

➢ Creation of an independent review panel to assess requirements for all formula components
➢ Modernize the definition of IF to reflect manufacturing realities and emphasize infant outcomes
➢ Evaluate the need to adjust iron content to align with current evidence
➢ Consider adding the fatty acids (DHA and AA) to the required nutrients
➢ Create a framework for the addition of bioactive components as evidence emerges
➢ Update regulations on quantity and quality of protein
➢ Incorporate energy density requirements
➢ Include specific regulations for formulas intended for preterm infants
➢ Reconsider labeling regulations to align more closely with international standards
➢ Evaluate concurrently the processes by which infant formulas are evaluated by the FDA before registration

AA, arachidonic acid; FFs, fatty acids; IF, infant formula.
